# A missing coronary guidewire mimicking aortic dissection—a case report

**DOI:** 10.1093/ehjcr/ytae100

**Published:** 2024-02-20

**Authors:** Ziad Arow, Avigdor Bar Sef, Abid Assali, Yoav Arnson

**Affiliations:** Cardiology Department, Meir Medical Center, Tchernichovsky St 59, Kfar Saba 4418001, Israel; Sackler Faculty of Medicine, Tel-Aviv University, Kiryat HaUniversita, Ramat Aviv Tel Aviv 6139001, Israel; Cardiology Department, Meir Medical Center, Tchernichovsky St 59, Kfar Saba 4418001, Israel; Sackler Faculty of Medicine, Tel-Aviv University, Kiryat HaUniversita, Ramat Aviv Tel Aviv 6139001, Israel; Cardiology Department, Meir Medical Center, Tchernichovsky St 59, Kfar Saba 4418001, Israel; Sackler Faculty of Medicine, Tel-Aviv University, Kiryat HaUniversita, Ramat Aviv Tel Aviv 6139001, Israel; Cardiology Department, Meir Medical Center, Tchernichovsky St 59, Kfar Saba 4418001, Israel; Sackler Faculty of Medicine, Tel-Aviv University, Kiryat HaUniversita, Ramat Aviv Tel Aviv 6139001, Israel

**Keywords:** Case report, Coronary guidewire, Cardiac computed tomography, Echocardiography, Aortic dissection

## Abstract

**Background:**

A retained coronary guidewire following coronary angiography is an extremely rare complication. We present a case of a retained coronary guidewire from a percutaneous coronary intervention done 2 years ago.

**Case summary:**

An 80-year-old asymptomatic man with a history of ischemic heart disease and moderate aortic stenosis presented to the echocardiography lab for routine follow-up. Transthoracic echocardiography showed Moderate aortic stenosis and a suspected linear echogenic structure in the ascending aorta. trans-esophageal echocardiography was performed to reveal a mobile and linear echogenic structure originating from the sinuses of Valsalva/Sinotubular junction and extending to the ascending aorta. An electrocardiogram gated cardiac computed tomography was performed and showed A linear well-defined structure originating from the ostium of the left main coronary artery and extending to the ascending aorta—a coronary guidewire from an earlier procedure. A second look at the last invasive coronary angiography record demonstrated the same finding. A multidisciplinary heart team discussion was obtained and concluded that the risk of surgical or endovascular intervention outweighed the potential benefit. The patient was discharged home for a close clinical and echocardiographic follow-up.

**Discussion:**

A retained coronary guidewire is a rare complication that operators should be aware of. Management should be case-specific depending on clinical presentation.

**Learning points**

- A retained coronary guidewire is a rare complication that operators should be aware of following percutaneous coronary intervention (PCI).
- The use of multi-modality imaging is essential for accurate diagnosis and a multidisciplinary heart team discussion is important for appropriate treatment decisions.

## Introduction

Invasive coronary angiography and percutaneous coronary intervention (PCI) have become the standard of care for all non-ST segment elevation myocardial infarction (NSTEMI) and ST-segment elevation myocardial infarction (STEMI) as well as for part of the patients with chronic coronary syndromes.^1,2,3^ Complications may occur during percutaneous coronary procedures or subsequently. Some of the common complications include coronary artery dissection or perforation, hyper-acute stent thrombosis, contrast-induced nephropathy or anaphylaxis, and vascular access site-related complications.^4,5^ A retained coronary guidewire is an extremely rare complication. We present a case of a retained coronary guidewire from a PCI done 2 years ago.

## Summary figure

**Figure:**
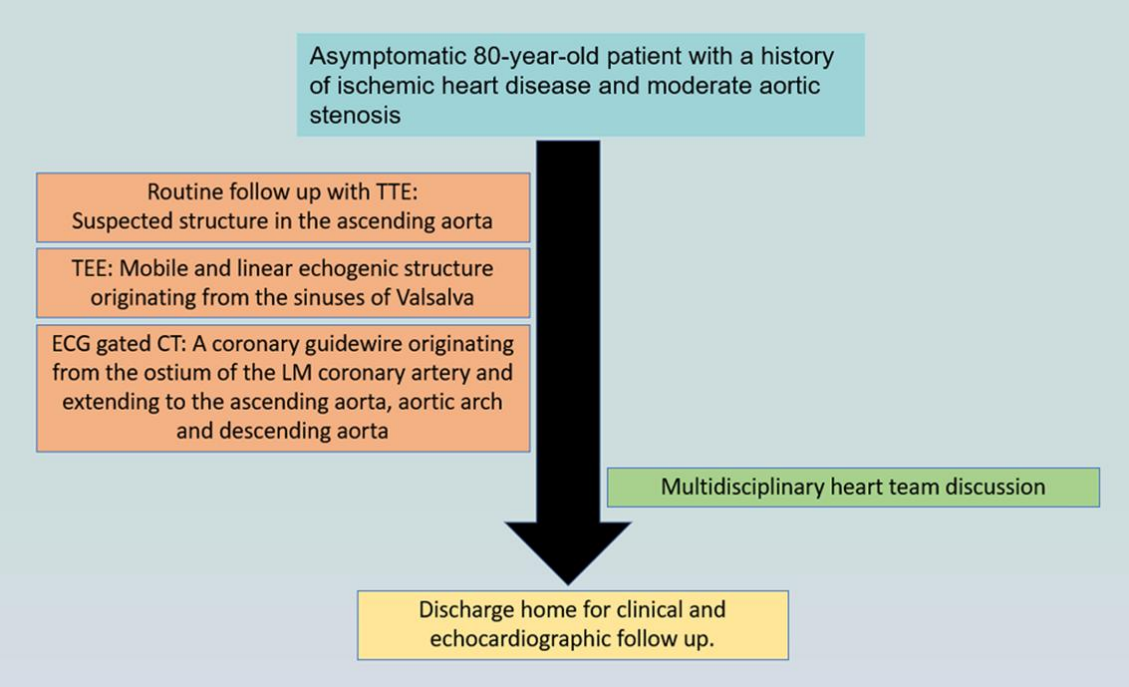


## Case presentation

An 80-year-old male presented to the echocardiography lab for routine follow-up for moderate aortic stenosis (AS). His medical comorbidities included hyperlipidaemia, hypertension, and type 2 diabetes mellitus. In the past, the patient underwent multiple PCI’s due to ischemic heart disease (IHD). His last coronary intervention involved implanting a drug eluting stent (DES) to the left main (LM) and proximal left anterior descending (LAD) coronary arteries, at an outside institution 2 years ago.

**Figure 1:**
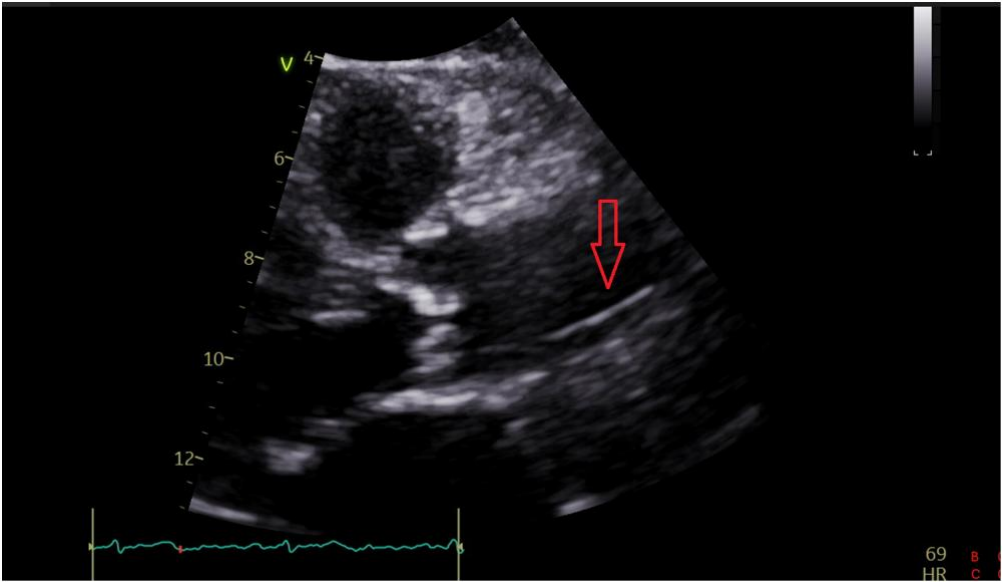
Transthoracic echocardiography.

**Figure 2:**
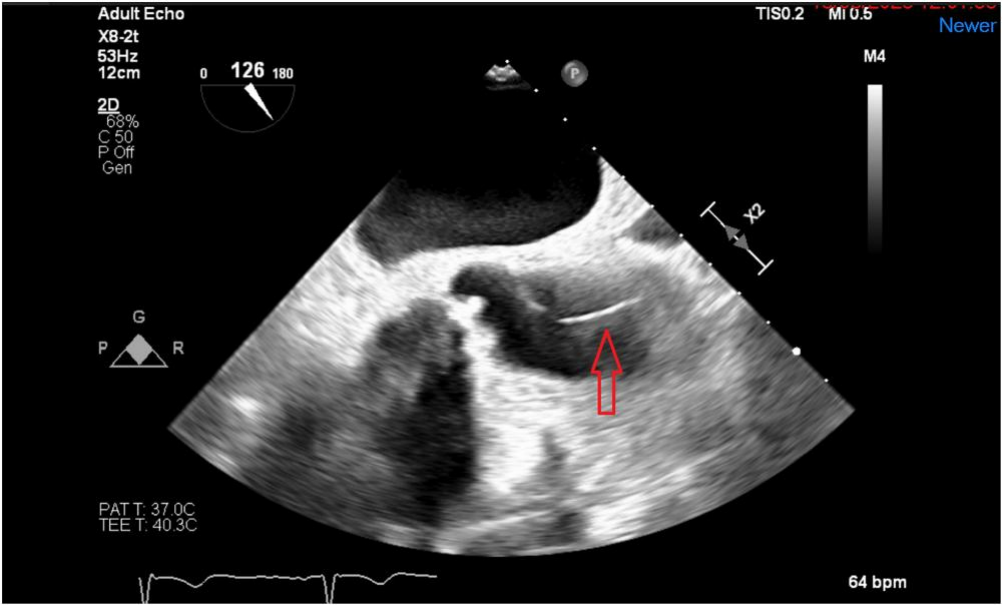
Trans-esophageal echocardiography.

**Figure 3:**
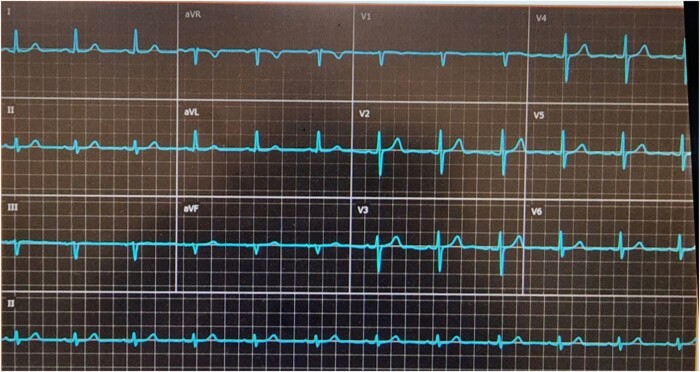
Twelve lead electrocardiogram.

**Figure 4:**
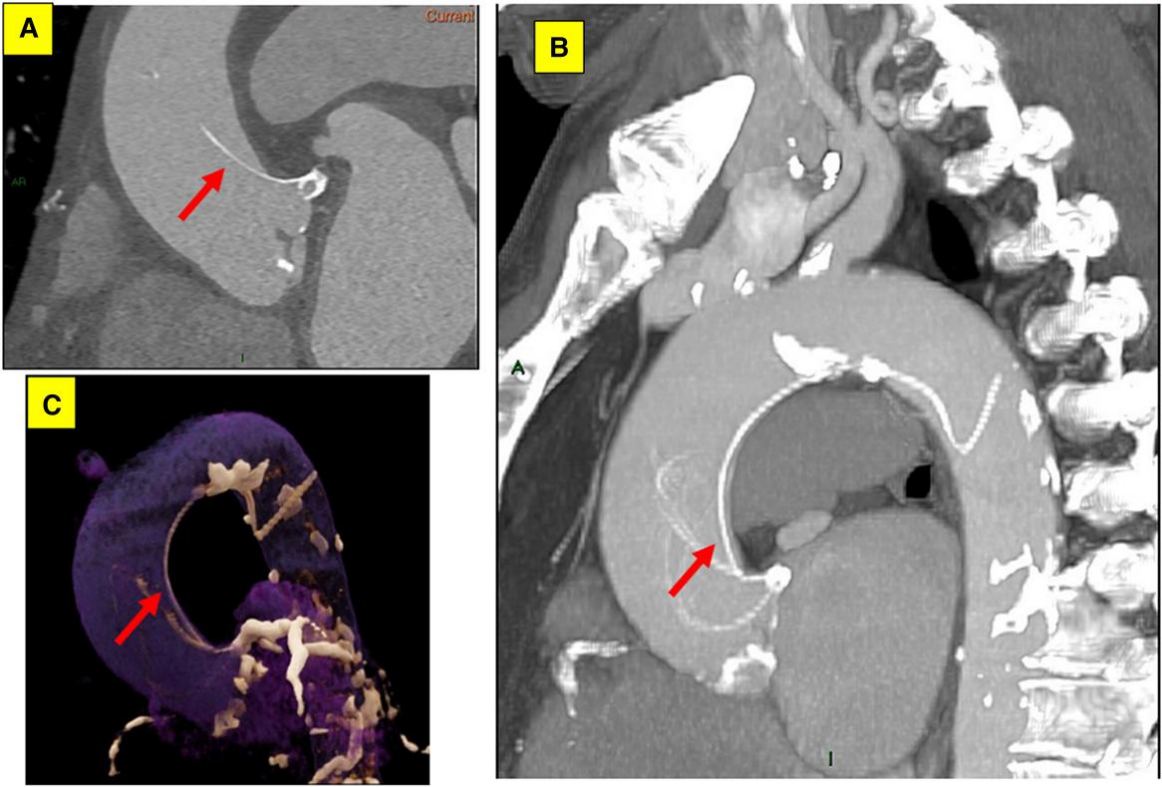
Electrocardiogram gated cardiac computed tomography.

The patient was hemodynamically stable and reported no chest pain, back pain, or dyspnoea. Cardiac auscultation demonstrated regular heart sounds with crescendo-decrescendo systolic murmur heard best at the right upper sternal border, radiating to the neck. His lungs were clear and he had no signs of heart failure. Initial Transthoracic echocardiography (TTE) showed normal left ventricular systolic function with moderate AS (aortic valve area: 1.1 cm², max and mean aortic gradients: 46 and 30 mmHg, respectively), moreover, a suspected linear echogenic structure was noted in the ascending aorta, which was suspicious for a dissection flap or an artefact (*Figure 1*, Supplementary material online, *Videos S1* and *S2*). As mentioned above, the patient was completely asymptomatic and for better visualization, evaluation, and characterization of the findings seen on TTE, trans-esophageal echocardiography (TEE) was immediately performed to reveal a mobile and linear echogenic structure originating from the sinuses of Valsalva/Sinotubular junction (STJ) and extending to the ascending aorta and the aortic arch (*Figure 2*, Supplementary material online, *Video S3*), highly suspicious for Stanford type A aortic dissection. No aortic regurgitation was noted (see Supplementary material online, *Video S4*). Electrocardiogram (ECG) showed normal sinus rhythm with no signs of acute ischaemia (*Figure 3*). Blood tests for high-sensitivity cardiac Troponin T (hs-cTnT) and Creatine Kinase-Myocardial Band (CK-MB) were negative. Immediate ECG-gated cardiac computed tomography (CT) was performed. In CT images the aortic endothel was intact and no false lumen was noticed, thus ruling out aortic dissection. A linear well-defined structure was noted originating from the ostium of the LM coronary artery and extending to the ascending aorta, aortic arch, and descending aorta, most probably a coronary guidewire from an earlier procedure (*Figure 4*). A second look at the last invasive coronary angiography record demonstrated the same finding at the end of the procedure—a coronary guidewire originating from the LM coronary artery and extending to the ascending aorta (see Supplementary material online, *Video S5*). A multidisciplinary heart team discussion, including an invasive cardiologist, a cardiothoracic surgeon, and a cardiac imaging specialist, was obtained, and concluded that the risk of surgical or endovascular intervention outweighed the potential benefit, particularly since the patient was completely asymptomatic, at advanced age and with multiple comorbidities. Discharge home followed several detailed explanations to the patient and his family regarding his condition and a recommendation for a close clinical and echocardiographic follow-up.

## Discussion

A retained coronary guidewire is an extremely rare complication that occurs in 0.1–0.2% of PCI procedures.^6^ In some cases of wire entrapment, over-rotation, or excessive bending—a wire fracture may occur.^7^ A retained coronary guidewire or guidewire remnants can lead to various complications including acute thrombosis or perforation of the coronary artery involved and systemic thrombo-embolization, leading to cerebrovascular accident (CVA).^8,9^

Most reported cases of entrapped guidewires were cases discovered during PCI.^10^ This is a rare case of a jailed wire behind the LM stent struts protruding the aorta, discovered years after the procedure. The use of multi-modality imaging was essential for accurate diagnosis and for further risk stratification. Management of patients with retained fractured guidewire fragments during PTCA remains particularly challenging due to the lack of data regarding clinical outcomes. There are no specific guidelines as to how this complication should be managed. Treatment options include conservative treatment and close follow-up (if the patient is completely asymptomatic), medical treatment with anticoagulation (in case of systemic embolization),^11^ and endovascular retrieval or surgical extraction (in case of acute thrombosis, perforation, or embolic phenomena threatening coronary artery occlusion).^10^

In conclusion, a retained coronary guidewire is a rare complication that operators should be aware of. Management should be case-specific depending on clinical presentation.

## Supplementary Material

ytae100_Supplementary_Data

## Data Availability

The data underlying this article will be shared on reasonable request to the corresponding author.

## Lead author biography

Ziad Arow is currently a resident in the cardiology department at Meir Medical Center in Israel. Ziad Arow finished his medical school (Technion, Israel) in 2016 and completed his internal medicine residency (Rabin Medical Center, Israel) in 2021.
